# Satisfaction With Life in IBS Is Associated With Psychological Burden Rather than Gastrointestinal Symptom Severity

**DOI:** 10.14309/ajg.0000000000002547

**Published:** 2023-10-04

**Authors:** Johanna T.W. Snijkers, Bjorn Winkens, Zsa Zsa R.M. Weerts, Lisa Vork, Zlatan Mujagic, Martine A.M. Hesselink, Carsten Leue, Joanna W. Kruimel, Jean W.M. Muris, Daisy M.A.E. Jonkers, Ad A.M. Masclee, Daniel Keszthelyi

**Affiliations:** 1 Division of Gastroenterology-Hepatology, Department of Internal Medicine, Maastricht University Medical Center+, Maastricht, the Netherlands;; 2 NUTRIM, School of Nutrition and Translational Research in Metabolism, Maastricht University, Maastricht, the Netherlands;; 3 Department of Methodology and Statistics, CAPHRI, Care and Public Health Research Institute, Maastricht University, Maastricht, the Netherlands;; 4 MHeNS, School for Mental Health and Neuroscience, Maastricht University, Maastricht, the Netherlands;; 5 Department of Psychiatry and Medical Psychology, Maastricht University Medical Center+, Maastricht, the Netherlands;; 6 Department of Family Medicine, CAPHRI Care and Public Health Research Institute, Maastricht University, Maastricht, the Netherlands.

**Keywords:** irritable bowel syndrome, life satisfaction, psychological factors, quality of life

## Abstract

**INTRODUCTION:**

Irritable bowel syndrome (IBS) has a major impact on emotional, social, and professional life. This study aimed to evaluate general life satisfaction, a subjective measure of well-being, in IBS patients, and to determine which factors are associated with higher life satisfaction.

**METHODS:**

IBS patients (n = 195, mean age 51.4 ± 16.5 years, 73.8% female) recruited from primary and secondary/tertiary care completed questionnaires regarding gastrointestinal symptoms, quality of life, psychological factors, and life satisfaction (Satisfaction With Life Scale, 5 items, range 5–35). A finite mixture model analysis was performed to identify latent classes. Multivariable linear regression was used to identify variables associated with life satisfaction.

**RESULTS:**

Overall, 71.3% of the patients were satisfied about their life (Satisfaction With Life Scale-score ≥21). Three latent subgroups could be identified with significantly higher life satisfaction in the subgroup with higher mental quality of life, fewer anxiety and depressive symptoms, lower gastrointestinal specific anxiety, and lower gastrointestinal symptom severity, compared with the other 2 groups. Multivariable linear regression showed that higher physical quality of life (B0.168, *P* < 0.001) and higher mental quality of life (B0.199, *P* < 0.001) were associated with higher life satisfaction. Using multivariable regression, no significant association was found between gastrointestinal symptom severity and life satisfaction.

**DISCUSSION:**

Higher physical and mental quality of life, but not gastrointestinal symptom severity, were independently associated with higher general life satisfaction in IBS. These findings reinforce the clinical need in IBS treatment to focus on the full extent of the disorder and not merely on gastrointestinal symptom improvement. ClinicalTrials.gov Identifier: NCT00775060.

## Graphical abstract

**Figure:**
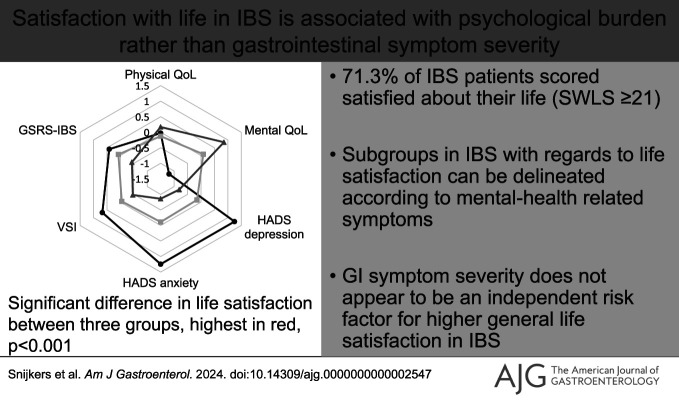

## INTRODUCTION

Irritable bowel syndrome (IBS) is a disorder of the gut-brain interaction, characterized by recurrent chronic abdominal pain and altered bowel habits (1). IBS has a global prevalence around 4% according to Rome IV criteria (2). IBS is associated with high disease burden: previous studies showed a significant decrease in (health-related) quality of life, increased healthcare utilization, and impact on daily activities (3–5). The professional life of an IBS patient is also affected by loss in work productivity (6). Furthermore, psychological comorbidities are often reported in patients with IBS (7,8). With this in mind, IBS symptoms affect a patient's life in different ways, and all these factors could influence patient's life satisfaction.

Life satisfaction is particularly relevant within the context of the current World Health Organization definition of health: “Health is a state of complete physical, mental, and social well-being and not merely the absence of disease or infirmity” (9). Well-being includes life satisfaction, which is about the individual's cognitive judgments considering their own values. This aspect makes it different from quality of life, whereby evaluation is based on particular criteria and domains using validated metrics. Life satisfaction, on the other hand, is more subjective and based on the factors the individual finds personally meaningful. This way it is possible that someone's life satisfaction is higher but quality of life lower compared with someone else because they may place importance on a different set of variables than those involved in quality of life (e.g., a homeless, disabled. or terminally ill person may have a higher life satisfaction than a wealthy person in good physical health). The most commonly used instrument for measuring life satisfaction is the 5-item Satisfaction With Life Scale (SWLS) (10), which has been used in different populations, including patients with Crohn's disease (11).

Previous research in IBS has extensively focused on quality of life, symptom severity, and treatment satisfaction. However, to the best of our knowledge, there are no studies available about general life satisfaction, as a dimension which is strongly influenced by subjective experiences, choices, priorities, and values of an individual. Therefore, we conducted exploratory analyses in IBS patients (Rome III) as part of a follow-up assessment after having been included in a cohort few years prior, to identify latent profiles in variables related to life satisfaction, and to ascertain which of these profiles and which variables were (independently) associated with higher life satisfaction. In addition, we also aimed to explore potential differences in a subgroup of patients by excluding those who did not fulfill the Rome III criteria at follow-up anymore, given the fluctuation nature of IBS symptoms.

## METHODS

### Study population

Data were obtained by the Maastricht Irritable Bowel Syndrome (MIBS) cohort study. The MIBS cohort was designed to identify etiological and pathophysiological factors in IBS patients. The study protocol had been approved by the Maastricht University Medical Center's Committee of Ethics (METC08-2-066) and registered in the US National Library of Medicine (NCT00775060). All study procedures were performed in compliance with Good Clinical Practice Guidelines and the revised Declaration of Helsinki. Written informed consent was given by each participant before participation.

Participants were recruited by the gastroenterology clinic of Maastricht University Medical Center, a secondary/tertiary referral hospital, and general practitioners in South-Limburg, the Netherlands. Patients with IBS diagnosed according to the Rome III criteria and aged 18–75 years were included. Further details of the study design of the MIBS cohort study have been described in detail elsewhere (12). Patient inclusion in the MIBS cohort was initiated in 2009. Patients who participated in the follow-up assessment of the MIBS cohort between September 2016 and March 2017 were eligible for inclusion in the current study. Only those patients who completed the SWLS questionnaire were included. A trained clinical investigator contacted all patients participating in this follow-up assessment and evaluated the Rome III diagnostic criteria during a telephonic interview. Not fulfilling the Rome criteria at follow-up did not lead to exclusion.

### Data collection

Data were collected by a self-reported questionnaire and included demographics, lifestyle factors, IBS subtype (Rome III criteria), treatment center, gastrointestinal (GI) symptoms, life satisfaction, quality of life, anxiety and depressive symptoms, and GI-specific anxiety.

Educational attainment was based on the Dutch educational system and then converted to the International Standard Classification of Education, including categories lower education, intermediate education, and tertiary education. The GI symptom score was determined using the Gastrointestinal Symptom Rating Scale-IBS (13 items, scale 1–7), higher scores indicating more severe symptoms (13). Satisfaction with life was obtained using the Satisfaction With Life Scale (SWLS, 5 items, scale 1–7). Subscores for the SWLS are classified as follows: 5–9 extremely dissatisfied, 10–14 dissatisfied, 15–19 slightly dissatisfied, 20 neutral, 21–25 slightly satisfied, 26–30 satisfied, and 31–35 extremely satisfied (10). Quality of life was based on the rand 36-item Short-Form Health Survey (scale 1–6), generating a physical and a mental health summary score, with higher scores indicating better quality of life (14,15). The Hospital Anxiety and Depression Scale (HADS, 14 items, scale 0–3) was used to assess anxiety and depressive symptoms, with higher scores indicating more severe anxiety and depressive symptoms (16). GI-specific anxiety was scored using the Visceral Sensitivity Index (15 items, scale 1–6), with higher scores indicating more severe GI-specific anxiety (17). All questionnaires used in the current study were collected at the time of the follow-up assessment.

### Statistical analysis

Statistical analyses were performed using IBM SPSS statistics for Windows, version 28.0.1.0 (IBM Corp., Armonk, NY) and R (version 4.0.5). The R package depmixS4 was used for the finite mixture model analysis (18). Baseline characteristics were analyzed using the independent samples *t* test for numerical variables and χ^2^ test for categorical variables. Numerical data are presented as means and SDs and categorical data as number of patients (%). A 2-sided *P*-value of ≤0.05 was considered statistically significant with correction for multiple testing according to Bonferroni-Holm.

A finite mixture model analysis was performed to identify latent profiles. This is a statistical strategy used to identify latent subgroups based on similar patterns of values. This was applied for the variables: GI symptom severity, anxiety symptoms, depressive symptoms, physical quality of life, mental quality of life, and GI-specific anxiety. Differences in SWLS between these 3 groups were tested with linear regression corrected for age, sex, and body mass index (BMI). The best model-fit was selected based on the lowest value of the Bayesian Information Criterion.

Subsequently, multivariable linear regression was performed to identify individual variables that were associated with higher satisfaction with life. The following parameters were included in the analyses: age, sex, BMI, smoking, alcohol intake, educational level, employment status, GI symptom severity, anxiety symptoms, depressive symptoms, physical quality of life, mental quality of life, and GI-specific anxiety. All assumptions for multivariable linear regression were assessed and met. These included histogram and Q-Q plot for normality; homoscedasticity, plots and variation, and Cook distance for outliers (cut-off 1); and applying a variance inflation factor for multicollinearity (variance inflation factor cut-off 10). Sensitivity analyses were performed in a subgroup of patients, e.g., only patients fulfilling Rome III criteria at follow-up. Multivariable logistic regression models were used to study the effect if the SWLS scores were dichotomized according to a cut-off value of ≥21. The backward elimination method (variable with largest *P*-value will be removed if *P* > 0.05) was applied as a sensitivity analysis. For the final model of this sensitivity analysis, the same Bonferroni-Holm correction as for the main analysis was used.

All authors had access to the study data and reviewed and approved the final manuscript.

## RESULTS

**Figure 1.**
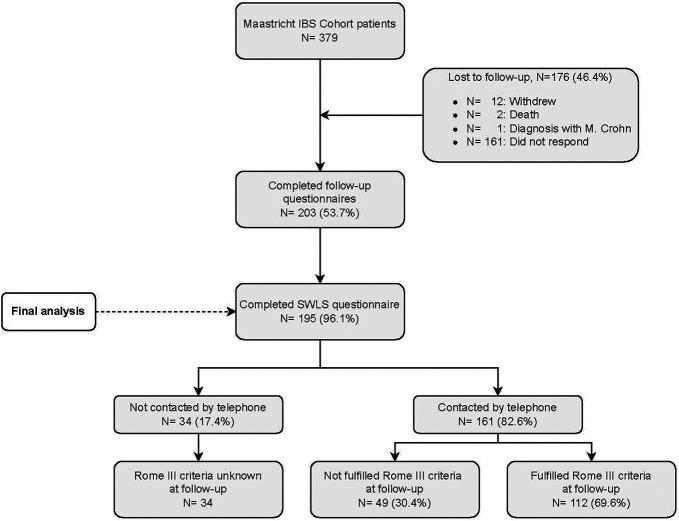
Flow chart participant selection.

Of the 379 patients invited, 195 patients completed the SWLS questionnaire and were included (shown in Figure 1). Of these, 161 patients could be reached by telephone to evaluate Rome III criteria for IBS, of whom 112 patients (69.6%) still met these criteria at the time of the follow-up measurement, i.e., approximately 5 years after initial inclusion.

Patients' characteristics are described in Table 1. Most patients were female (73.8%), and mean age was 51.44 (SD 16.5) years. Patients who still fulfilled the Rome III criteria at follow-up (n = 112) differed significantly from those who did not fulfill the Rome III criteria anymore (n = 49) at follow-up regarding the following variables: gastrointestinal symptoms (*P* < 0.001), anxiety symptoms (*P* = 0.003), and GI-specific anxiety (*P* < 0.001). The other variables in the characteristics table, in particular the SWLS score (*P* = 0.078), did not significantly differ.

**Table 1.**
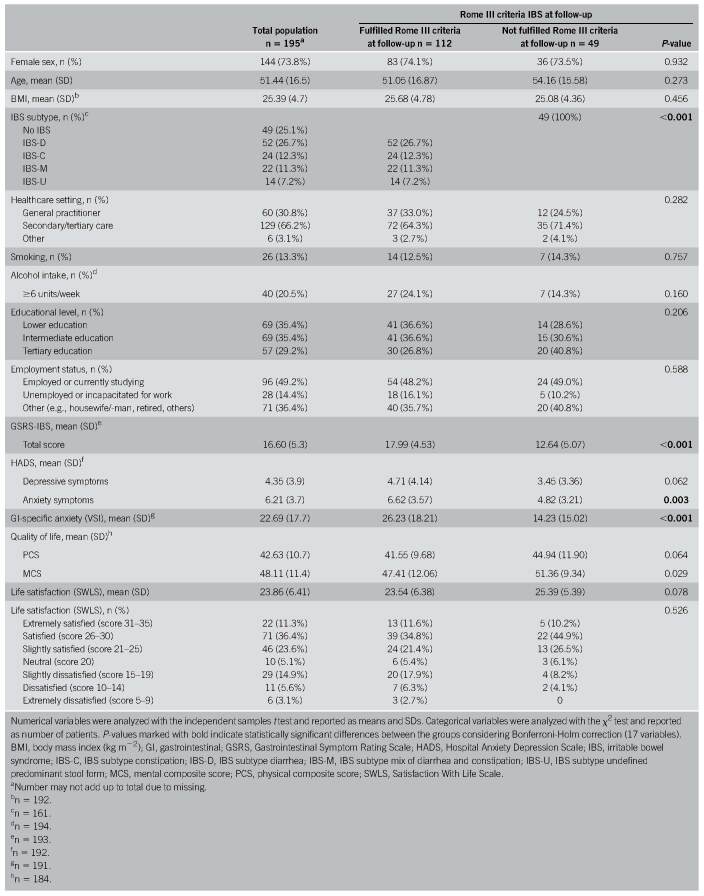
Characteristics of the total study population and separated for Rome III positive and negative patients at follow-up

There were 139 patients (71.3%) with a SWLS-score ≥21 corresponding with (slightly-extremely) satisfied life satisfaction and 56 patients (28.7%) had a SWLS-score <21 corresponding with neutral or (slightly-extremely) dissatisfied life satisfaction. Differences between these 2 groups (satisfied versus dissatisfied) are presented in Supplementary Table 1 (see Supplementary Digital Content, http://links.lww.com/AJG/D79). The nonresponders for the follow-up assessment (n = 184) were significantly younger compared with the responders (n = 195) (40.26 years versus 47.35 years, *P* < 0.001), see Supplementary Table 2, see Supplementary Digital Content, http://links.lww.com/AJG/D79.

### Finite mixture model analysis

**Figure 2.**
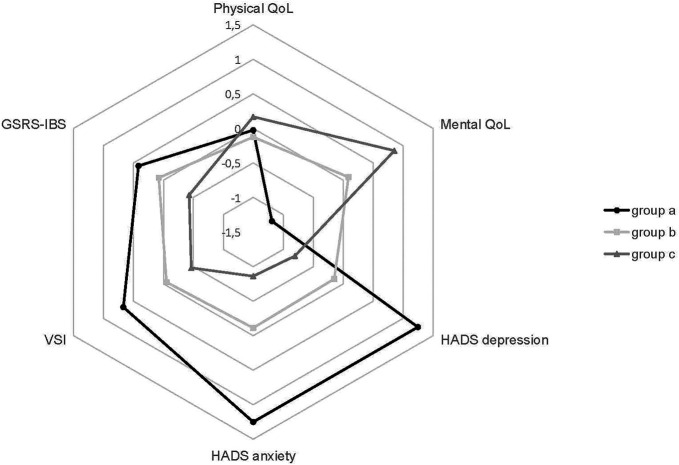
Profiles of the 3 subgroups identified in the irritable bowel syndrome cohort by finite mixture model analysis. The z-score is calculated in relation to the total cohort average and SD.

The model with 3 latent subgroups resulted in the best model-fit (shown in Supplementary Figure 1, Supplementary Digital Content, http://links.lww.com/AJG/D79). Group *a* was characterized by a below average score for mental quality of life and above average scores for depressive symptoms, anxiety symptoms, GI-specific anxiety, and GI symptom severity. Group *b* was characterized by average scores for all variables. Group *c* was the opposite of group *a* with an above average score for mental quality of life and below average scores for depressive symptoms, anxiety symptoms, GI-specific anxiety, and GI symptom severity. This is demonstrated in Figure 2 while their mean (SD) scores on the individual variables are presented in Supplementary Table 3 (see Supplementary Digital Content, http://links.lww.com/AJG/D79).

These 3 groups significantly differed in scores for mental quality of life, general anxiety symptoms, depressive symptoms, GI-specific anxiety, and GI symptom severity (*P* < 0.001 for all variables) corrected for age, sex, and BMI (shown in Supplementary Table 4, see Supplementary Digital Content, http://links.lww.com/AJG/D79). Physical quality of life did not differ significantly (*P* = 0.076) between the 3 groups. GI symptom severity was the lowest in group *c*, and there were more subjects included in group *c* who did not fulfill the Rome III criteria at follow-up anymore (38.1%) compared with the other 2 groups (15.4% in group *a* and 21.3% in group *b*). Differences between the 3 groups in satisfaction with life were significant (*P* < 0.001), with the highest life satisfaction in group *c*.

### Multivariable regression model

Linear regression model was used which included age, sex, BMI, alcohol, smoking, educational level, employment, GI symptom severity, anxiety symptoms, depressive symptoms, physical quality of life, mental quality of life, and GI-specific anxiety. A correlation matrix for these last 6 variables and SWLS is presented in Supplementary Table 5 (see Supplementary Digital Content, http://links.lww.com/AJG/D79). This model showed higher life satisfaction for higher physical quality of life (B 0.168, 95% confidence interval [CI] 0.085; 0.251, *P* < 0.001), higher mental quality of life (B 0.199, 95% CI 0.096; 0.302, *P* < 0.001), and not being unemployed (B −3.233, 95% CI −5.481; −0.984, *P* = 0.005) (shown in Table 2). Employment status did not reach statistical significance when correction for multiple testing was applied.

**Table 2.**
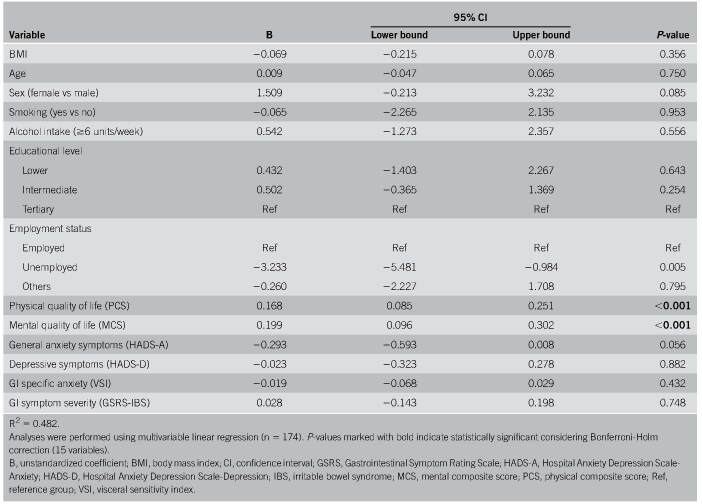
Results of multivariable linear regression analysis for satisfaction with life (SWLS) in an IBS population

When backward elimination was used, variables associated with higher life satisfaction were higher physical quality of life (*P* < 0.001), higher mental quality of life (*P* < 0.001), fewer anxiety symptoms (*P* = 0.020), female sex (*P* = 0.014), and not being unemployed (*P* = 0.001). Considering correction for multiple testing, female sex and fewer anxiety symptoms were not statistically significant (shown in Supplementary Table 6, see Supplementary Digital Content, http://links.lww.com/AJG/D79).

The same linear regression model was applied to a subgroup of patients for whom Rome III criteria were confirmed at follow-up (n = 112). In this subgroup, there were no significant predictors for higher life satisfaction after correction for multiple testing (data not shown). Without correction for multiple testing, the same significant variables as in the total group were found.

As additional sensitivity analysis, data were also analyzed with a dichotomized SWLS-score as outcome. Logistic regression analyses were performed according to a cut-off value of ≥21 for satisfaction. There were no significant predictors for higher life satisfaction after correction for multiple testing. Without correction for multiple testing, variables which were significantly associated with a satisfied score were higher mental quality of life, higher physical quality of life, not being unemployed, and female sex (shown in Supplementary Table 7, see Supplementary Digital Content, http://links.lww.com/AJG/D79).

## DISCUSSION

In this study, we demonstrated that 71% of the patients with IBS were satisfied about their life (SWLS-score ≥21). Finite mixture model analysis identified latent subgroups which with regard to life satisfaction could be delineated according to mental health-related symptoms. Multivariable linear regression analysis investigating the contribution of different factors revealed further that higher life satisfaction was associated with higher physical and mental quality of life. Not being unemployed, female sex, and fewer general anxiety symptoms might play a role in higher life satisfaction but were not significant when correcting for multiple testing. Interestingly, gastrointestinal symptom severity was not an independent risk factor for life satisfaction.

This is, to the best of our knowledge, the first study that examined general life satisfaction in an IBS patient population. We found a mean (±SD) of the SWLS-score of 23.9 ± 6.4 in our IBS cohort. Two previous studies demonstrated higher SWLS-scores in the general population (mean 25.6 ± 5.3 in men, 25.7 ± 5.8 in women) (19) and in healthy young adults (26.18 ± 5.72) (20). Lower SWLS-scores were demonstrated in patients with active Crohn's disease (mean 20.8 ± 8.6 in men, 20.9 ± 7.8 in women) (11), patients with psychiatric disorders (mean 20.1 ± 7.8) (21), type 2 diabetes mellitus (mean 20.0 ± 5.0) (22), and in Parkinson disease (mean 21.6 ± 7.0 in men, 20.4 ± 6.2 in women) (23). Our results are comparable with a study by Sarid et al with similar SWLS-scores in patients with Crohn's disease in remission (23.8 ± 6.4 in men, 23.8 ± 6.7 in women) (11).

Life satisfaction is a dimension of well-being that has hitherto received little attention within the context of IBS. Traditionally, outcomes in both clinical practice and trials have focused on gastrointestinal symptoms and more specifically abdominal pain, which was also driven by current guidelines and recommendations from regulatory authorities (24,25). More recently, increased emphasis has been given to quality of life and the impact on mental health. It is not unsurprising that we here show that the quality of life has a strong relationship with life satisfaction in IBS, in line with findings from other populations (26,27). However, it is important to recognize the difference between life satisfaction and quality of life. One may perceive their quality of life as similar to one's compatriots and yet may still feel dissatisfied. The SWLS is a measure of the judgmental component of subjective well-being (28), i.e., individuals evaluate the satisfaction with their lives based on their own values and experiences, and therefore, SWLS also measures a degree of acceptance with one's life circumstances. The degree of integrating health status in the SWLS questionnaire is determined by the individual and, therefore, carries an important subjective element.

Life satisfaction as a separate, integrative, and subjective dimension is particularly relevant as healthcare delivery is increasingly being framed with the concept of positive health including 6 different domains: bodily functions, mental well-being, meaningfulness, quality of life, social and societal participation, and daily functioning (29). In this respect, it is imperative to understand and to be aware of factors influencing life satisfaction to increase the added value of healthcare delivered beyond mere reduction of gastrointestinal symptom burden.

To ascertain factors driving life satisfaction, we used finite mixture model analysis and identified different subgroups within our study population which provides more insight into the patterns of scores across the variables. Life satisfaction was significantly higher in the subgroup with higher mental quality of life, less anxiety and depressive symptoms, lower GI-specific anxiety, and lower GI symptom severity. The magnitude of the difference between the subgroups was the greatest for mental quality of life, depressive symptoms, anxiety symptoms, and gastrointestinal-specific anxiety. These findings support the clinical need to take psychological factors into account and the importance of integrating mental healthcare in IBS treatment as they appear most relevant for life satisfaction.

When several factors were analyzed individually using multiple regression, a noticeable finding in our study was that gastrointestinal symptom severity was not significantly associated with life satisfaction. We have previously reported that decrease in GI symptom burden is not parallel by an increase in quality of life (30). As the SWLS-questionnaire assesses the judgment of one's life as a whole and does not measure satisfaction in specific life domains, patients determine themselves how to integrate their (GI and non-GI) symptoms in answering these questions. Rather, a number of other factors appear to play a more important role in being satisfied.

These other factors may include not being unemployed, female sex, and fewer general anxiety symptoms, although statistical significance was not reached for these factors considering correction for multiple testing. Regarding employment status, paid employment provides direct access to resources and contributes to an individual's meaningfulness and participation in the society which may contribute to higher life satisfaction (31). Although a potential association between sex and life satisfaction is not clear in the literature (11,20,32), a possible explanation for the higher life satisfaction in women are the better emotional coping strategies in women than men (11).

Strengths of this study include using a validated questionnaire to evaluate life satisfaction for the first time in an IBS population, which made it possible to compare life satisfaction with other (chronic) disorders.

A limitation of the current study is the use of data of a follow-up measurement rather than the initial time of IBS diagnosis. First, not all patients filled in the questionnaires or were reached by telephone interview to confirm the retention of the diagnosis of IBS, which could reflect a selection bias. In addition, due to the fluctuating nature of IBS symptoms (33) and the fact that the follow-up was performed several years after initial inclusion, 30.4% of the included participants no longer met the Rome III criteria for IBS at follow-up and we were unable to establish the presence of the diagnosis in an additional 17.4%. When linear regression was performed in the group of patients for whom Rome III criteria were confirmed at follow-up, we no longer found any statistically significant predictors. We believe that this is because of the smaller sample size (just over half of the total population), resulting in lower power, as the same stringency in correction for multiple testing was also applied for this subgroup. This is supported by the fact that no difference in life satisfaction was found between patients who fulfilled the Rome III criteria at follow-up vs those who did not. On the other hand, even patients who no longer fulfilled the Rome III criteria at follow-up did so at the time of initial inclusion in the cohort and, therefore, including this population allows further insight into IBS characteristics. The fact that the Rome classification relies only on symptoms while life satisfaction does not might also explain the same SWLS scores in patients not fulfilling Rome criteria anymore.

Moreover, the development of symptom-related coping strategies could also explain why factors closely related to the disorder itself become less relevant over time. It could very well be the case that other factors associated with life satisfaction which were not assessed in this study, such as household income and composition, religion and spiritual beliefs, as well as cultural values, could be of relevance in this respect. In addition, cultural differences relating to socioeconomic factors including social policies of (vocational) rehabilitation, the organization of (mental) healthcare, and public attitude toward people with disorders such as IBS can also contribute to life satisfaction. These factors deserve to be investigated further, and for this reason, our findings from the Netherlands might not readily be extrapolated to other sociocultural contexts or healthcare delivery systems. In addition, this sample may not represent correlates of life satisfaction among nonhealthcare seekers.

Similarly to the above, the lack of significant results when dichotomizing the SWLS outcome might be due to the loss in power as dichotomizing a numerical outcome usually results in a loss of information. Besides this, the current study consisted of 1 time point, and no data were available about received treatment, including potential dietary adjustments, and fluctuation in life satisfaction over time.

For the same reason, analyses performed here only allow to assess for the presence of association but do not advise on causality. Therefore, future studies should be of longitudinal nature to understand the dynamics of different contributing factors.

Furthermore, the diagnostic criteria applied in this study were based on the Rome III consensus and, therefore, cannot readily be extrapolated to populations defined according to different standards, i.e., Rome IV criteria.

In conclusion, mental quality of life seems an important determinant of life satisfaction in IBS and allows identification of subgroup associated with high life satisfaction. In addition, higher mental and physical quality of life were independently associated with a higher life satisfaction in patients with IBS. Interestingly, no association was found with gastrointestinal symptom severity, when assessed independently from other factors. These findings reinforce the clinical need in IBS treatment to focus not merely on gastrointestinal symptom improvement without attention for the full extent of the disorder. Our data suggest further that clinical outcomes related to life satisfaction, as measured by the easily administered 5-item SWLS, may serve as a useful additional endpoint to measure patient well-being.

## CONFLICTS OF INTEREST

**Guarantor of the article:** D. Keszthelyi, MD, PhD.

**Specific author contributions:** J.T.W.S.: study concept and design, data analysis and interpretation, manuscript writing. B. W.: study concept and design, data analysis, constructive review of manuscript. Z.Z.R.M.W.: study design cohort, data collection, constructive review of manuscript. L.V.: study design cohort, data collection, constructive review of manuscript. Z.M.: study design cohort, data collection, constructive review of manuscript. M.A.M.H.: study design cohort, data collection and processing, constructive review of manuscript. C.L.: study design cohort, constructive review of manuscript. J.W.K.: study design cohort, constructive review of manuscript. J.W.M.M.: study design cohort, constructive review of manuscript. D.M.A.E.J.: study design cohort, constructive review of manuscript. A.A.M.M.: study design cohort, constructive review of manuscript. D.K.: study concept and design, data interpretation, constructive review of manuscript. All authors approved the final manuscript.

**Financial support:** None to report.

**Potential competing interests:** Z.Z.R.M.W. has received funding from WillPharma to attend a scientific meeting. Z.M. reports grants form Niels Stensen Fellowship, MLDS, ZonMw, and Galapagos and has received advisory board fees from Johnson and Johnson (paid to host institution) and speaker's fee (paid to host institution) from Galapagos, all outside the submitted work. MAMH and DMAEJ are partly financed by Public-Private partnerships Grants of Top Knowledge Institute “Well on Wheat” and “Well on Wheat 2.0”. Part of the work of DMAEJ is financed by the Carbokinetics program as part of the NWO-CCC Partnership Program, by Organic A2BV/Mothersfinest BV, by EU grants FP7 SysmedIBD/305564, BIOM/305479 and Character/305676, COST action GENIEUR, and H2020 DISCOvERIE/848228. J.W.M.M. received funding from ZonMw and Rome Foundation. A.A.M.M. has received research funding from Dutch Cancer Society, Will Pharma, and ZonMw. D.K. has received research funding from Grunenthal, Allergan, Will Pharma, UEG, MLDS, Rome Foundation, ZonMw, Horizon Europe, Horizon 2020. D.K. has received speaker's fee (paid to host institution) from Dr Falk. J.T.W.S., B.W., L.V., C.L., J.W.K. have nothing to disclose.

**Data availability statement:** The data that support the findings of this study are available from the corresponding author, JTW Snijkers, on reasonable request.

**Study Highlights**

**WHAT IS KNOWN**

✓ IBS is associated with high disease burden, which results in substantial impact on the life of affected individuals. There are no studies available about general life satisfaction in the IBS population.

**WHAT IS NEW HERE**

✓ 71.3% of IBS patients scored satisfied about their life.
✓ Subgroups in IBS regarding life satisfaction can be delineated according to mental health-related symptoms.
✓ Gastrointestinal symptom severity does not seem to be an independent risk factor for higher general life satisfaction in IBS.
✓ Life satisfaction should be considered as an auxiliary outcome to healthcare delivery in IBS.

## Supplementary Material

## References

[1] Lacy BE Mearin F Chang L Bowel disorders. Gastroenterology 2016;150(6):1393–407.e5. 10.1053/j.gastro.2016.02.031 27144627

[2] Oka P Parr H Barberio B . Global prevalence of irritable bowel syndrome according to Rome III or IV criteria: A systematic review and meta-analysis. Lancet Gastroenterol Hepatol 2020;5(10):908–17. 32702295 10.1016/S2468-1253(20)30217-X

[3] Gralnek IM Hays RD Kilbourne A . The impact of irritable bowel syndrome on health-related quality of life. Gastroenterology 2000;119(3):654–60. 10982758 10.1053/gast.2000.16484

[4] Ballou S Keefer L. The impact of irritable bowel syndrome on daily functioning: Characterizing and understanding daily consequences of IBS. Neurogastroenterology Motil 2017;29(4). doi: 10.1111/nmo.12982. PMC5367953 27781332

[5] Levy RL Von Korff M Whitehead WE Costs of care for irritable bowel syndrome patients in a health maintenance organization. Am J Gastroenterol 2001;96(11):3122–9. 11721759 10.1111/j.1572-0241.2001.05258.x

[6] Silk DB. Impact of irritable bowel syndrome on personal relationships and working practices. Eur J Gastroenterol Hepatol 2001;13(11):1327–32. 11692059 10.1097/00042737-200111000-00011

[7] Fond G Loundou A Hamdani N Anxiety and depression comorbidities in irritable bowel syndrome (IBS): A systematic review and meta-analysis. Eur Arch Psychiatry Clin Neurosci 2014;264(8):651–60. 24705634 10.1007/s00406-014-0502-z

[8] Drossman DA Chang L Bellamy N Severity in irritable bowel syndrome: a Rome Foundation Working Team Report. Am J Gastroenterol 2011;106(10):1749–59; quiz 1760. 21747417 10.1038/ajg.2011.201

[9] WHO. Basic documents: Forty-ninth edition (including amendments adopted to 31 may 2019); Geneva 2020.

[10] Diener E. Understanding Scores on the Satisfaction with Life Scale, 2006.

[11] Sarid O Slonim-Nevo V Pereg A Coping strategies, satisfaction with life, and quality of life in Crohn's disease: A gender perspective using structural equation modeling analysis. PLoS One 2017;12(2):e0172779. 28245260 10.1371/journal.pone.0172779 PMC5330481

[12] Mujagic Z Jonkers D Ludidi S Biomarkers for visceral hypersensitivity in patients with irritable bowel syndrome. Neurogastroenterology Motil 2017;29(12). doi: 10.1111/nmo.13137. 28675524

[13] Wiklund IK Fullerton S Hawkey CJ An irritable bowel syndrome-specific symptom questionnaire: Development and validation. Scand J Gastroenterol 2003;38(9):947–54. 14531531 10.1080/00365520310004209

[14] McHorney CA Ware JE Jr Raczek AE. The MOS 36-item Short-Form Health Survey (SF-36): II. Psychometric and clinical tests of validity in measuring physical and mental health constructs. Med Care 1993;31(3):247–63. 8450681 10.1097/00005650-199303000-00006

[15] Farivar SS Cunningham WE Hays RD. Correlated physical and mental health summary scores for the SF-36 and SF-12 Health Survey, V.I. Health Qual Life Outcomes 2007;5:54. 17825096 10.1186/1477-7525-5-54 PMC2065865

[16] Zigmond AS Snaith RP. The hospital anxiety and depression scale. Acta psychiatrica Scand 1983;67(6):361–70. 10.1111/j.1600-0447.1983.tb09716.x 6880820

[17] Labus JS Bolus R Chang L The visceral sensitivity index: Development and validation of a gastrointestinal symptom-specific anxiety scale. Aliment Pharmacol Ther 2004;20(1):89–97. 10.1111/j.1365-2036.2004.02007.x 15225175

[18] Visser I Speekenbrink M. depmixS4: An R package for hidden markov models. J Stat Softw 2010;36(7):1–21.

[19] van Loon AM Tijhuis M Surtees PG . Personality and coping: Their relationship with lifestyle risk factors for cancer. Personal Individual Differences 2001;31(4):541–53.

[20] Arrindell WA Heesink J Feij JA. The satisfaction with life scale (SWLS): Appraisal with 1700 healthy young adults in The Netherlands. Personal Individual Differences 1999;26(5):815–26.

[21] Arrindell WA van Nieuwenhuizen C Luteijn F. Chronic psychiatric status and satisfaction with life. Personal Individual Differences 2001;31(2):145–55.

[22] Brzoza KB Główczyński P Piegza M Acceptance of the disease and quality of life in patients with type 1 and type 2 diabetes. Eur J Psychiatry 2022;36:114–9.

[23] Lucas-Carrasco R Den Oudsten BL Eser E . Using the satisfaction with life scale in people with Parkinson's disease: A validation study in different European countries. Scientific World J 2014;2014:680659. 10.1155/2014/680659 PMC3929569 24672353

[24] U.S. Department of Health and Human Services Food and Drug Administration CDER. Guidance for Industry Irritable Bowel Syndrome—Clinical Evaluation of Drugs for Treatment, 2012.

[25] European Medicines Agency CHMP. Guideline on the Evaluation of Medicinal Products for the Treatment of Irritable Bowel Syndrome, 2014.

[26] Yildirim Y Kilic SP Akyol AD. Relationship between life satisfaction and quality of life in Turkish nursing school students. Nurs Health Sci 2013;15(4):415–22. 23336720 10.1111/nhs.12029

[27] Kulczycka L Sysa-Jedrzejowska A Robak E. Quality of life and satisfaction with life in SLE patients-the importance of clinical manifestations. Clin Rheumatol 2010;29(9):991–7. 20532577 10.1007/s10067-010-1509-0 PMC2908751

[28] Pavot W Diener E. The Satisfaction with Life Scale and the emerging construct of life satisfaction. J Positive Psychol 2008;3(2):137–52.

[29] iPH. Institute of Positive Health. Dialogue Tool 2.0. iph.nl/en, 2022.

[30] Weerts Z Vork L Mujagic Z Reduction in IBS symptom severity is not paralleled by improvement in quality of life in patients with irritable bowel syndrome. Neurogastroenterology Motil 2019;31(8):e13629. 10.1111/nmo.13629 PMC6852246 31119844

[31] Warr P. Well-being and the workplace. In: Well-being: The foundations of Hedonic Psychology. Russell Sage Foundation: New York, NY, 1999, pp 392–412.

[32] Clench-Aas J Nes RB Dalgard OS . Dimensionality and measurement invariance in the satisfaction with life scale in Norway. Qual Life Res 2011;20(8):1307–17. 21308414 10.1007/s11136-011-9859-x PMC3178031

[33] Ford AC Forman D Bailey AG . Irritable bowel syndrome: A 10-yr natural history of symptoms and factors that influence consultation behavior. Am J Gastroenterol 2008;103(5):1229–39; quiz 1240. 18371141 10.1111/j.1572-0241.2007.01740.x

