# Supplementary material for: Satisfaction With Life in IBS Is Associated With Psychological Burden Rather than Gastrointestinal Symptom Severity
Source: Am J Gastroenterol. 2023 Oct 4;119(3):512–20. doi: 10.14309/ajg.0000000000002547 (PMC10904003; doi:10.14309/ajg.0000000000002547)
Supplement: Supplementary file 1 [file acg-119-512-s001.docx]

**Supplementary**

Supplementary Figure 1. BIC values plotted for number of subgroups by finite mixture model analysis.
Lowest BIC value indicates the optimum number of subgroups, this is achieved for three subgroups.

Supplementary Table 1. Characteristics of the study population according to a satisfied life satisfaction (SWLS ≥21, n=139) and dissatisfied life satisfaction (SWLS <21, n=56)

|  | **Satisfied according to SWLS** | | |
| --- | --- | --- | --- |
|  | Satisfied (SWLS ≥21)  n=139 | Dissatisfied  (SWLS <21)  n=56 | *p*-value |
| Female sex, n (%) | 111 (79.9%) | 33 (58.9%) | **0.003** |
| Age, mean (SD) | 51.02 (16.7) | 52.48 (16.0) | 0.577 |
| BMI, mean (SD) ^†a^ | 25.12 (4.8) | 26.1 (4.5) | 0.191 |
| Fulfilled Rome III criteria at follow-up, n (%) ^†b^  Yes  No | 76 (54.7%)  40 (28.8%) | 36 (64.3%)  9 (16.1%) | 0.073 |
| IBS subtype, n (%) ^†b^  No IBS  IBS-D  IBS-C  IBS-M  IBS-U | 40 (28.8%)  34 (24.5%)  17 (12.2%)  17 (12.2%)  8 (5.8%) | 9 (16.1%)  18 (32.1%)  7 (12.5%)  5 (8.9%)  6 (10.7%) | 0.263 |
| Healthcare setting, n (%)  General practitioner  Secondary/tertiary care  Other | 44 (31.7%)  93 (66.9%)  2 (1.4%) | 16 (28.6%)  36 (64.3%)  4 (7.2%) | 0.188 |
| Smoking, n (%) | 17 (12.2%) | 9 (16.1%) | 0.475 |
| Alcohol intake, n (%) ^†c^  ≥6 units/week | 29 (20.9%) | 11 (19.6%) | 0.893 |
| Educational level, n (%)  Lower education  Intermediate education  Tertiary education | 46 (33.1%)  47 (33.8%)  46 (33.15) | 23 (41.1%)  22 (39.3%)  11 (19.6%) | 0.171 |
| Employment status, n (%)  Employed or currently studying  Unemployed or incapacitated for work  Other (e.g. housewife/-man, retired, other) | 78 (56.1%)  11 (7.9%)  50 (36.0%) | 18 (32.1%)  17 (30.4%)  21 (37.5%) | **<0.001** |
| GSRS-IBS, mean (SD) ^†d^  Total score  Abdominal pain  Bloating  Constipation  Diarrhea  Satiety | 15.98 (5.3)  3.60 (1.5)  3.88 (1.5)  2.61 (1.6)  2.97 (1.4)  2.94 (1.5) | 18.17 (4.9)  4.04 (1.4)  4.06 (1.3)  2.91 (1.7)  3.39 (1.5)  3.84 (1.6) | 0.009 |
| HADS, mean (SD) ^†e^  Depressive symptoms  Anxiety symptoms | 3.34 (2.9)  5.30 (3.0) | 6.94 (5.0)  8.49 (4.2) | **<0.001**  **<0.001** |
| GI-specific anxiety, mean (SD) ^†f^ | 20.26 (17.0) | 28.85 (18.2) | **0.002** |
| Quality of life, mean (SD) ^†g^  PCS  MCS | 44.20 (10.0)  51.30 (8.7) | 38.84 (11.4)  40.41 (13.4) | **0.002**  **<0.001** |

Numerical variables were analyzed with independent samples t-test and reported as means and standard deviations. Categorical variables were analyzed with the Chi-square test and reported as number of patients. P-values marked with bold indicate statistically significant differences between the groups considering Bonferroni-Holm correction (16 variables).
Abbreviations: SWLS, Satisfaction With Life Scale; IBS, Irritable Bowel Syndrome; BMI, Body Mass Index (kg m^-2^); GSRS, Gastrointestinal Symptom Rating Scale; HADS, Hospital Anxiety Depression Scale; GI, gastrointestinal; PCS, Physical Composite Score; MCS, Mental Composite Score; IQR, interquartile range; n, number of patients.
^†^Number may not add up to total due to missing, ^†a^n=192, ^†b^n=161, ^†c^n=194, ^†d^n=193, ^†e^n=192, ^†f^n=191, ^†g^n=184.

Supplementary Table 2. Characteristics of the study population at time of initial inclusion, responders (n=195) versus non-responders (n=184) for the follow-up assessment

|  | **Participation follow-up assessment** | | |
| --- | --- | --- | --- |
|  | Responders  n=195 | Non-responders  n=184 | *p*-value |
| Female sex, n (%) | 145 (74.4%) | 129 (70.1%) | 0.355 |
| Age, mean (SD) | 47.35 (16.39) | 40.26 (16.49) | **<0.001** |
| BMI, mean (SD) ^†a^ | 24.86 (4.94) | 25.03 (4.69) | 0.753 |
| IBS subtype, n (%)  IBS-D  IBS-C  IBS-M  IBS-U | 68 (34.9%)  39 (20%)  78 (40%)  10 (5.1%) | 55 (29.9%)  34 (18.5%)  86 (46.7%)  9 (4.9%) | 0.606 |
| Healthcare setting, n (%)  General practitioner  Secondary/tertiary care  Other | 60 (30.8%)  129 (66.2%)  6 (3.1%) | 35 (19%)  145 (78.8%)  4 (2.2%) | 0.022 |
| Smoking, n (%)^†b^ | 33 (16.9%) | 45 (24.5%) | 0.015 |
| Alcohol intake, n (%) ^†c^  ≥6 units/week | 27 (13.8%) | 21 (11.4%) | 0.733 |
| GSRS, mean (SD) ^†d^  Total score* | 3.12 (0.82) | 3.34 (0.94) | 0.026 |

Numerical variables were analyzed with independent samples t-test and reported as means and standard deviations. Categorical variables were analyzed with the Chi-square test and reported as number of patients. P-values marked with bold indicate statistically significant differences between the groups considering Bonferroni-Holm correction (8 variables).
Abbreviations:; IBS, Irritable Bowel Syndrome; BMI, Body Mass Index (kg m^-2^); GSRS, Gastrointestinal Symptom Rating Scale; HADS, Hospital Anxiety Depression Scale; n, number of patients.
^†^Number may not add up to total due to missing, ^†a^n=332, ^†b^n=335, ^†c^n=326, ^†d^n=325

* GSRS total score is the average of all five sub-scores: abdominal pain, reflux syndrome, diarrhea syndrome, constipation syndrome, and indigestion syndrome. GSRS-IBS score was not available at time of initial inclusion

Supplementary Table 3. Profiles of the three subgroups identified in the IBS cohort by finite mixture model analysis.

|  | **Group a**  **(n=52)** | **Group b**  **(n=80)** | **Group c**  **(n=63)** |
| --- | --- | --- | --- |
| **Age** | 52.65 ± 16.04 | 50.06 ± 17.21 | 52.19 ± 16.02 |
| **BMI** | 25.36 ± 4.64 | 25.26 ± 4.62 | 25.60 ± 4.88 |
| **Female sex** | 31 (59.6%) | 64 (80.0%) | 49 (77.78%) |
| **Physical quality of life (PCS)** | 42.33 ± 10.17 | 41.29 ± 11.21 | 44.43 ± 10.43 |
| **Mental quality of life (MCS)** | 34.54 ± 10.58 | 49.11 ± 5.99 | 57.89 ± 3.24 |
| **General anxiety symptoms**  **(HADS-A)** | 10.82 ± 3.35 | 5.80 ± 1.69 | 3.03 ± 1.48 |
| **Depressive symptoms (HADS-D)** | 9.26 ± 3.87 | 3.78 ± 2.01 | 1.17 ± 0.93 |
| **GI specific anxiety (VSI)** | 34.57 ± 17.71 | 21.78 ± 16.04 | 14.48 ± 14.66 |
| **GI symptom severity**  **(GSRS-IBS)** | 18.79 ± 3.79 | 17.00 ± 5.45 | 14.33 ± 5.36 |
|  |  |  |  |
| **IBS subtype**  **Unknown**  **No IBS**  **IBS-D**  **IBS-C**  **IBS-M**  **IBS-U** | 9 (17.3%)  8 (15.4%)  16 (30.8%)  10 (19.2%)  7 (13.5%)  2 (3.8%) | 16 (20.0%)  17 (21.3%)  23 (28.8%)  9 (11.3%)  6 (7.5%)  9 (11.3%) | 9 (14.3%)  24 (38.1%)  13 (20.6%)  5 (7.9%)  9 (14.3%)  3 (4.8%) |
| **Smoking** | 7 (13.5%) | 8 (10%) | 11 (17.5%) |
| **Alcohol intake (≥6units/week)** | 11 (21.2%) | 16 (20%) | 13 (20.6%) |
| **Educational level**  **Lower**  **Intermediate**  **Tertiary** | 22 (42.3%)  20 (38.5%)  10 (19.2%) | 26 (32.5%)  28 (35.0%)  26 (32.5%) | 21 (33.3%)  21 (33.3%)  21 (33.3%) |
| **Employment status**  **Employed**  **Unemployed**  **Other** | 23 (44.2%)  9 (17.3%)  20 (38.5%) | 39 (48.8%)  13 (16.3%)  28 (35.0%) | 34 (54.0%)  6 (9.5%)  23 (36.5%) |
| **Satisfaction with life (SWLS)** | 18.88 ± 6.30 | 24.45 ± 5.57 | 27.21 ± 4.88 |

Numerical data expressed as mean ± standard deviation (SD). Categorical data expressed as number of patients with percentage of subgroup (subgroup a, b, or c).

Supplementary Table 4. Differences between the three subgroups identified in the IBS cohort by finite mixture model analysis

| **Variable** | **Estimated Mean (SE)** | **B (95%CI)** | ***p*-value** |
| --- | --- | --- | --- |
| **Physical quality of life (PCS)**  Group a  Group b  Group c | 43.253 (1.499)  41.641 (1.320)  45.738 (1.400) | -2.485 (-6.427;1.457)  -4.097 (-7.642;-0.553)  - | 0.076 (overall)  0.215  **0.024**  - |
| **Mental quality of life (MCS)**  Group a  Group b  Group c | 34.506 (0.999)  48.855 (0.880)  57.420 (0.933) | -22.914 (-25.541;-20.286)  -8.565 (-10.927;-6.202)  - | **<0.001** (overall)  **<0.001**  **<0.001**  - |
| **General anxiety symptoms (HADS-A)**  Group a  Group b  Group c | 10.965 (0.361)  5.917 (0.268)  3.171 (0.295) | 7.794 (6.958;8.630)  2.746 (2.017;3.475)  - | **<0.001** (overall)  **<0.001**  **<0.001**  **-** |
| **Depressive symptoms (HADS-D)**  Group a  Group b  Group c | 9.194 (0.348)  3.911 (0.295)  1.255 (0.323) | 7.939 (7.024;8.854)  2.656 (1.857;3.455)  - | **<0.001** (overall)  **<0.001**  **<0.001**  **-** |
| **GI specific anxiety (VSI)**  Group a  Group b  Group c | 35.087 (2.358)  22.483 (1.978)  14.895 (2.177) | 20.191 (13.991;26.392)  7.587 (2.208;12.966)  - | **<0.001** (overall)  **<0.001**  **0.006**  **-** |
| **GI symptom severity (GSRS-IBS)**  Group a  Group b  Group c | 18.596 (0.712)  16.473 (0.611)  13.776 (0.666) | 4.820 (2.938;6.703)  2.697 (1.043;4.352)  - | **<0.001** (overall)  **<0.001**  **0.002**  **-** |
| **Satisfaction with life (SWLS)**  Group a  Group b  Group c | 18.911 (0.779)  23.676 (0.673)  26.636 (0.737) | -7.725 (-9.801;-5.650)  -2.960 (-4.787;-1.134)  - | **<0.001** (overall)  **<0.001**  **0.002**  - |

Analyses were performed using linear regression corrected for age, sex, and body mass index. P-values marked with bold indicate statistically significant considering Bonferroni-Holm correction (7 variables).
Abbreviations: B, unstandardized coefficient; SE, standardized error; CI, confidence interval; PCS, physical composite score; MCS, mental composite score; HADS-A, hospital anxiety depression scale-anxiety; HADS-D, hospital anxiety depression scale-Depression; VSI, visceral sensitivity index; GSRS-IBS, Gastrointestinal Symptom Rating Scale- Irritable Bowel Syndrome.

Supplementary Table 5. Correlation matrix

|  | **Satisfaction with life scale (SWLS)** | **Physical quality of life (PCS)** | **Mental quality of life (MCS)** | **General anxiety symptoms (HADS-A)** | **Depressive symptoms (HADS-D)** | **GI specific anxiety (VSI)** | **GI symptom severity (GSRS-IBS)** |
| --- | --- | --- | --- | --- | --- | --- | --- |
| **Satisfaction with life scale (SWLS)** | 1 |  |  |  |  |  |  |
| **Physical quality of life (PCS)** | 0.332 | 1 |  |  |  |  |  |
| **Mental quality of life (MCS)** | 0.517 | -0.099 | 1 |  |  |  |  |
| **General anxiety symptoms (HADS-A)** | -0.484 | 0.053 | -0.727 | 1 |  |  |  |
| **Depressive symptoms (HADS-D)** | -0.498 | -0.119 | -0.755 | 0.720 | 1 |  |  |
| **GI specific anxiety (VSI)** | -0.290 | -0.221 | -0.299 | 0.380 | 0.344 | 1 |  |
| **GI symptom severity (GSRS-IBS)** | -0.264 | -0.280 | -0.268 | 0.289 | 0.222 | 0.476 | 1 |

Analyses were performed using Pearson correlation coefficients. Abbreviations: IBS, Irritable Bowel Syndrome; PCS, physical composite score; MCS, mental composite score; HADS-A, hospital anxiety depression scale-anxiety; HADS-D, hospital anxiety depression scale-Depression; VSI, visceral sensitivity index; GSRS, Gastrointestinal Symptom Rating Scale. Variation Inflation Factor for all analyses was <5.

ho

Supplementary Table 6. Results of multivariable linear regression analysis using backward elimination for satisfaction with life (SWLS) in an IBS population

| **Variable** | **B** | **95% CI** | | ***p*-value** |
| --- | --- | --- | --- | --- |
|  |  | **Lower Bound** | **Upper Bound** |  |
| **Sex (female versus male)** | 1.954 | 0.406 | 3.502 | 0.014 |
| **Physical quality of life (PCS)** | 0.185 | 0.115 | 0.255 | **<0.001** |
| **Mental quality of life (MCS)** | 0.203 | 0.117 | 0.289 | **<0.001** |
| **General anxiety symptoms (HADS-A)** | -0.311 | -0.573 | -0.049 | 0.020 |
| **Employment status**  **Employed**  **Unemployed**  **Other** | Ref.  -3.462  0.257 | Ref.  -5.573  -1.291 | Ref.  -1.352  1.806 | **-**  **0.001**  0.743 |

R^2^= 0.483

Analyses were performed using multivariable linear regression backward elimination by hand. P-values marked with bold indicate statistically significant considering Bonferroni-Holm correction (15 variables).
Abbreviations: B, unstandardized coefficient; SE, standardized error; CI, confidence interval; Ref, reference group; BMI, body mass index; PCS, physical composite score; MCS, mental composite score; HADS-A, hospital anxiety depression scale-anxiety; HADS-D, hospital anxiety depression scale-Depression; VSI, visceral sensitivity index

Supplementary Table 7. Results of multivariable logistic regression analysis for SWLS≥21

| **Variable** | **OR** | **95% CI** | | ***p*-value** |
| --- | --- | --- | --- | --- |
|  |  | **Lower Bound** | **Upper Bound** |  |
| **BMI** | 0.940 | 0.863 | 1.024 | 0.157 |
| **Age** | 0.997 | 0.962 | 1.032 | 0.853 |
| **Sex (Female versus Male)** | 3.044 | 1.044 | 8.873 | 0.041 |
| **Physical quality of life (PCS)** | 1.053 | 1.001 | 1.106 | 0.044 |
| **Mental quality of life (MCS)** | 1.076 | 1.015 | 1.140 | 0.014 |
| **General anxiety symptoms (HADS-A)** | 0.899 | 0.749 | 1.079 | 0.253 |
| **Depressive symptoms (HADS-D)** | 0.998 | 0.834 | 1.195 | 0.986 |
| **GI specific anxiety (VSI)** | 0.995 | 0.968 | 1.024 | 0.744 |
| **GI symptom severity (GSRS-IBS)** | 0.983 | 0.881 | 1.097 | 0.761 |
| **Smoking (yes versus no)** | 0.532 | 0.147 | 1.920 | 0.335 |
| **Alcohol intake (≥6units/week)** | 2.524 | 0.757 | 8.416 | 0.132 |
| **Educational level**  **Lower**  **Intermediate**  **Tertiary** | Ref.  1.258  1.213 | Ref.  0.436  0.384 | Ref.  3.629  3.835 | 0.904  -  0.672  0.742 |
| **Employment status**  **Employed**  **Unemployed**  **Other** | Ref.  0.197  0.603 | Ref.  0.054  0.181 | Ref.  0.717  2.005 | 0.047  -  0.014  0.373 |

Analyses were performed using multivariable logistic regression, variables included: age, sex, BMI, GI symptom severity, anxiety symptoms, depressive symptoms, physical quality of life, mental quality of life, and GI specific anxiety. Abbreviations: OR, odds ratio; SE, standardized error; CI, confidence interval; BMI, body mass index; PCS, physical composite score; MCS, mental composite score; HADS-A, hospital anxiety depression scale-anxiety; HADS-D, hospital anxiety depression scale-Depression; VSI, visceral sensitivity index; GSRS-IBS, Gastrointestinal Symptom Rating Scale- Irritable Bowel Syndrome; GI, gastrointestinal.
